# Multiple Systemic Arterial Aneurysms in Kawasaki Disease

**DOI:** 10.7759/cureus.42714

**Published:** 2023-07-31

**Authors:** Sara Hamwi, Mohamad B Alebaji, Ahmed E Mahboub, Eiman H Alkaabi, Najla S Alkuwaiti

**Affiliations:** 1 Pediatric Medicine, Tawam Hospital, Al Ain, ARE; 2 Pediatrics, Tawam Hospital, Al Ain, ARE

**Keywords:** pediatrics emergency, uae, systemic artery aneurysms (saa), coronary aneurysms, kawasaki disease

## Abstract

Kawasaki disease is a self-limiting systemic vasculitis that can lead to various cardiac complications, including coronary dilatation and aneurysms. However, systemic aneurysms are uncommon and only occur in rare cases. In this instance, we present the case of an eight-week-old infant who presented to the emergency department with fever, loose motion, and neck swelling, ultimately diagnosed with Kawasaki disease accompanied by multiple systemic arterial aneurysms. This case highlights the potential for Kawasaki disease to cause systemic aneurysms and emphasizes the importance of recognizing and monitoring this rare complication in patients diagnosed with Kawasaki disease.

## Introduction

Kawasaki disease (KD) is an acute, self-limited vasculitis of unknown etiology that primarily affects children younger than five years old. It was first described in 1967 by Dr. Tomisaku Kawasaki and has since become the most common cause of acquired heart disease in children in developed countries [1]. KD typically presents with fever, rash, lymphadenopathy, conjunctivitis, lips, oral cavity erythema, and changes to the extremities, such as edema or desquamation [2]. Despite the vast majority of patients fully recovering, a small subset develops systemic artery aneurysms (SAA) [3].

SAA is a rare but severe KD complication associated with an increased risk of myocardial infarction, stroke, and death. It is usually seen in the coronary arteries [3]. However, it may also occur in the carotid, vertebral, renal, and mesenteric arteries [4]. The pathogenesis of SAA is not fully understood; however, it is believed to result from a combination of inflammation, thrombus formation, and endothelial damage [4].

This case report describes an eight-week-old infant diagnosed with Kawasaki disease and had developed systemic artery aneurysms (SAAs). The purpose of this report is to present the clinical course, management, and outcome of this rare complication of KD.

## Case presentation

An eight-week-old male infant presented to the emergency department, having been unwell for the preceding six days with fever, neck swelling, and bloody stools containing mucus. His past medical and birth histories were unremarkable, and he had received all his vaccinations. On the first day of illness, he was reluctant to feed and was taken to a local hospital, where he was admitted and treated for a presumed neck abscess with IV antibiotics. On the second day, he developed loose stools mixed with fresh blood, associated with abdominal distention. His stool culture was positive for Entamoeba histolytica and Clostridium difficile A toxin. He was then transferred to our facility for further investigations. On arrival, he was active, alert, afebrile, and well-hydrated, with a SpO2 of 99% at room air, blood pressure of 90/65 mmHg, and a heart rate of 150 beats per minute. There was mild abdominal distention, and he was grunting and mildly tachypneic. He had right-sided neck lymph node enlargement. The rest of the examination was unremarkable. Initial investigations (Table 1) showed a low hemoglobin of 8.1 mg/dl, leucocytosis of 24.84 x 10^9^/L, thrombocytopenia of 104 x 10^9^/L, and elevated inflammatory markers, with C-reactive protein (CRP) of 147.9 mg/L and procalcitonin (PCT) of 2.17 ng/mL.

**Table 1:** Laboratory investigations in the first admission

Investigations	Lab result	Reference range
Albumin	14 g/L	28-47 g/L
Ferritin	2480.8 pmol/L	31-1454 pmol/L
Interleukin-6	111.1 pg/mL	< 7 pg/mL
NT-pro BNP	13,486 ng/mL	< 320 ng/mL
Troponin-T	12.0 ng/mL	< 14 ng/mL
Procalcitonin	2.17 ng/mL	< 0.5 ng/mL
White blood count (WBC)	24.8 x 10^9^/L	< 15.0 x 10^9^/L
Hemoglobin	81 g/L	94-112 g/L
Neutrophils	20.56 x 10^9^/L	1-8.5 x 10^9^/L
Prothrombin time (PT)	14.9 seconds	11.5-15.3 seconds
International normalized ratio (INR)	1.41	0.86-1.22
Activated partial thromboplastin time (aPTT)	59.3 seconds	35.1-46.3
D-Dimer	15.4 mg/L	0.11-0.42 mg/L
C-Reactive Protein	147.9 mg/L	< 5 mg/L

**Figure 1:**
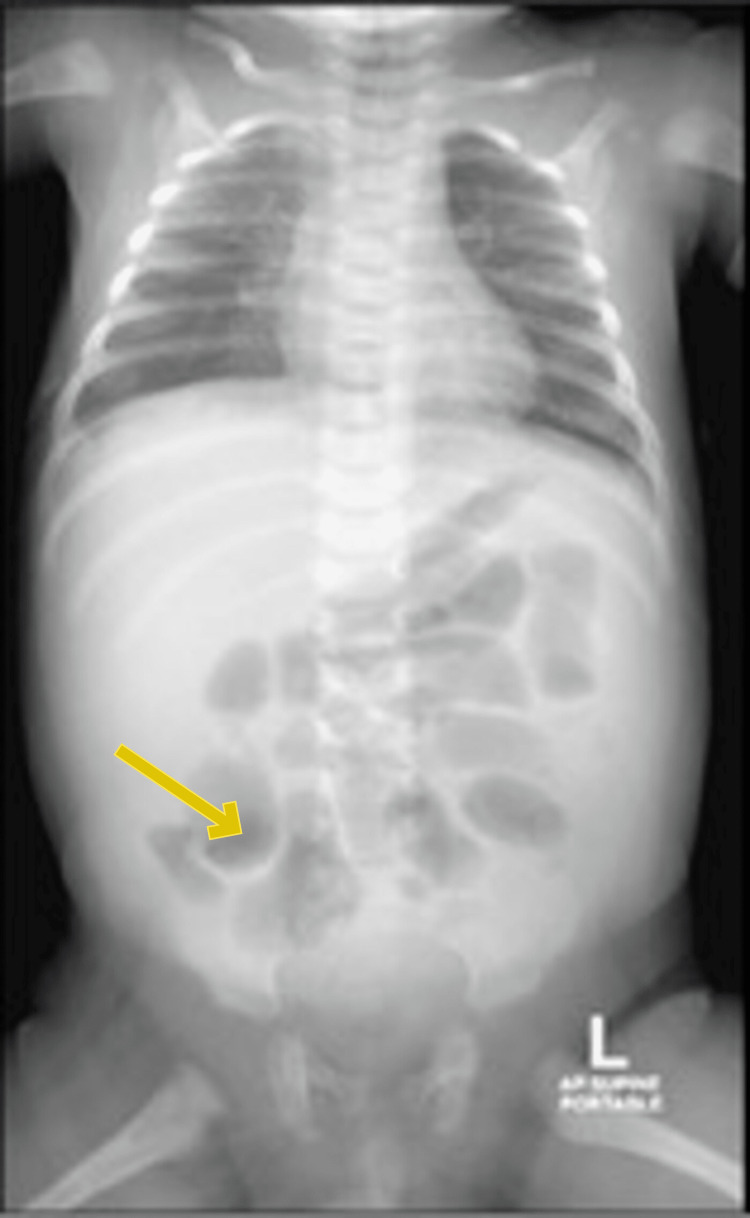
X-ray showing increased bronchovascular markings, and gut gases

An X-ray revealed a mild increase in bronchovascular markings, while an abdominal revealed small bowel loops that were slightly distended and gas-filled (Figure 1). An ultrasound showed minimal free ascitic fluid and right pleural effusion.

The results of the clinical and paraclinical tests were suggestive of a systemic illness. He was started on IV antibiotics, Cefotaxime, Ampicillin, and Metronidazole, and was also given a packed RBC transfusion for his anemia. In addition, his history included conjunctivitis, hepatomegaly, and direct hyperbilirubinemia, which could suggest viral infection, metabolic disorders, liver disease, and possible immunodeficiency. His repeated investigation showed a hemoglobin level of 8.5 mg/dl, worsening leucocytosis of 54 x 10^9^/L, thrombocytosis of 934 x 10^9^/L, low fibrin, and high D-Dimers. SARS antibodies, lactate dehydrogenase, ferritin, CRP, PCT, Interleukin-6, and Interleukin-2 levels were all elevated. Flow cytometry was done to rule out immunodeficiency, which showed a low count and percentages of CD3, CD4, CD8, and CD28, as well as a high CD19 and standard NK and a high CD4/CD8 ratio.

Based on clinical and laboratory findings, he was treated as a case of Multisystem Inflammatory Syndrome in Children (MIS-C) and started on intravenous immunoglobulin (IVIG), high-dose steroids, and aspirin. He improved clinically and remained afebrile, active, tolerating feeds, with regular bowel movements and urine output. His enlarged right cervical lymph node resolved. He was then sent home with a tapering dose of steroids and aspirin.

Five days after discharge, he was readmitted with a productive cough, purulent eye discharge, nasal congestion, and high-grade fever. His inflammatory markers were still persistently elevated (Table 2).

**Table 2:** Laboratory investigations in the second admission ESR: Erythrocyte sedimentation rate; PT: Prothrombin time; INR: International normalized ratio; aPTT: Activated partial thromboplastin time

Investigations	Lab result	Reference range
Albumin	24 g/L	28-47 g/L
Ferritin	1725.8 pmol/L	31-1454 pmol/L
NT-pro BNP	1,740 ng/mL	< 320 ng/mL
Troponin-T	7.8 ng/mL	< 14 ng/mL
Procalcitonin	0.54 ng/mL	< 0.5 ng/mL
WBC	21.4 x 10^9^/L	< 15.0 x 10^9^/L
Hemoglobin	73 g/L	94-112 g/L
ESR	78 mm/hr	0-20 mm/hr
PT	10.4 seconds	11.5-15.3 seconds
INR	0.97	0.86-1.22
aPTT	32.5 seconds	35.1-46.3
D-Dimer	9.87 mg/L	0.11-0.42 mg/L
Fibrinogen	8.10 g/L	0.82-3.83 g/L
C-Reactive Protein	208.5 mg/L	< 5 mg/L

**Figure 2:**
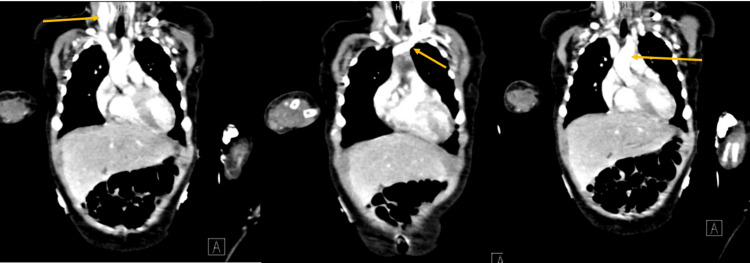
CT angiography showing multiple aneurysms

A lumbar puncture was performed with the parent's consent to rule out sepsis, which came back unremarkable. Echocardiography confirmed the diagnosis of Kawasaki Disease (KD) by revealing massive aneurysms in the right coronary artery (RCA), left main coronary artery (LMCA), left anterior descending artery (LAD), and RCA. CT angiography images (Figure 2) revealed a left external carotid artery aneurysm.

Multiple focal dilatations/ectasias were also seen in axillary and brachial, vertebral, and brachiocephalic arteries. Mural irregularity in the aortic arch, descending thoracic aorta, and abdominal aorta showed distal tapering and narrowing together with ectasia and tortuosity of mediastinal and esophageal branches; the latter formed an incomplete ring around the distal esophagus. Beading was observed with multiple dilatations and narrowing of the coeliac trunk, renal, splenic, inferior mesenteric, and left external femoral arteries, accompanied by a focal dilatation of the SMA origin. Dilatation of both common iliac arteries just below the bifurcation was also seen, along with dilatation and tortuosity of both internal iliac arteries.

Based on these findings, he was diagnosed with a case of Kawasaki disease with multiple systemic arterial aneurysms. He was started on triple anti-coagulation, methylprednisolone, and biological agents (Anakinra). Our patient had been diagnosed with a particularly severe type of Kawasaki illness.

## Discussion

Systemic artery aneurysms in children are uncommon but can occur alongside infection, trauma, connective tissue disorders, congenital vascular abnormalities, or vasculitis [5]. Two percent of untreated KD patients develop systemic artery aneurysms [6]. Few cases report systemic artery aneurysms in KD [5, 7, 8]. The pathogenesis is still not fully understood. It is postulated that interleukins have a significant role in developing aneurysms [9], particularly IL-6. According to a literature review, few extra coronary medium-sized artery involvements have been observed in Kawasaki's disease. As with this patient, systemic arterial aneurysms caused by KD were virtually exclusively reported within the first eight months of life [6]. According to previous research, subclavian, brachial, axillary, and iliac arteries are the most prevalent sites for developing SAAs [10].

The patient's young age at diagnosis is one of the risk factors predisposing to systemic problems. Infants identified before six months are more prone than older individuals to develop coronary aneurysms [11]. It has been established that early diagnosis and treatment with IVIG and aspirin reduce the risk of coronary aneurysm development from 25% to 3% to 5% [12]. Additionally, our case was treated with IVIG and steroids. Aneurysms' consequences are progression to rupture, thrombosis, and embolic complications [13]. Fortunately, our patient did not get any of the abovementioned complications.

## Conclusions

This case demonstrates the significance of maintaining a high suspicion index for systemic arterial aneurysms in KD patients. CT or magnetic resonance imaging of systemic arteries, such as the brachial and iliac arteries, should also be explored as a screening technique.
